# The evolution of HIV-1 subtypes and disease progression among adults on anti-retroviral drugs in Northern Tanzania

**DOI:** 10.1186/1742-4690-9-S2-P366

**Published:** 2012-09-13

**Authors:** E Shao, B Nyombi, J Sabuni

**Affiliations:** 1 Kilimanjaro Clinical Research Institute (KCRI)-Moshi Tanzania, Moshi, United Republic of Tanzania

## Background

The human immunodeficiency virus (HIV) type 1 epidemic in African countries is mainly due to HIV-1 Subtype C compared to United States of America (USA) and Europe where mainly is B. However many scientists remain in the dark regarding disease progression of these Subtypes and their influence on the clinical manifestation of HIV infection. Prospectively we looked at the association between HIV-1 subtypes A, B, C, D and non-reactive viruses as well as the rate of disease progression in a longitudinal study of seropositive adults from Northern Tanzania.

## Methods

A total of 104 adults were selected from PRIOR-6 study aiming on ARV adherence among HIV/AIDS patients on ART in Moshi Kilimanjaro for HIV-1 subtypes and characterization. The CD4+ cell counts were was measured at baseline and at three, six and twelve months. The chi-square and odds ratios were used for the analysis to measure the association between viral subtype and the rate of disease progression.

## Results

Relative to patients with subtypes C, patients with subtype D experienced the most rapid progression to World Health Organization clinical stage 4 with odds ratio of 12 and 95% confidence interval (CI), 1.67–68.56) and to a CD4+ cell count of <200 cells/mm3 during our one year follow up.

## Conclusion

We noticed heterogeneity in the rates of disease progression of HIV-1 disease in infected people, basing on the infecting subtype. Even at the baseline CD4+ count patients with Subtype D had the very low count followed by C and A was the least. Subtype D was associated with the most rapid progression of the disease, compared to the subtype A, or C of viruses in our study.

